# Efficacy of Recombinant Adenoviral Human p53 Gene in Treatment of Malignant Pleural or Peritoneal Effusions

**DOI:** 10.3779/j.issn.1009-3419.2013.03.07

**Published:** 2013-03-20

**Authors:** Xin ZHANG, Yi HU, Jinliang WANG, Sujie ZHANG, Haitao TAO, Sun JING, Baishou WU

**Affiliations:** 1 Department of Oncology, the General Hospital of Chinese PLA, Beijing 100853, China; 2 National Key Lab of Molecular Oncology, Cancer Institute & Hospital, CAMS, Beijing 100021, China

**Keywords:** *p53* gene, Gene therapy, Malignant effusion, Intra-cavity infusion

## Abstract

**Background and objective:**

Once the malignant pleural or peritoneal effusion is developed it is difficult to control. This report presents a new method for controlling the malignant effusions.

**Methods:**

Forty-eight patients, 29 males and 19 females with an average age of 61.2 years old, who were satisfied with the study inclusion criteria, were recruited in this study. Twenty-seven and 21 patients had a malignant pleural and peritoneal effusion, respectively. After draining most of fluids, these patients received intra-cavity infusion of rAd-*p*53 once per week for 4 weeks, at dose of 2×10^12^ viral particles (VP) diluted into 200 mL of saline solution for pleural effusions, and 4×10^12^ VP diluted into 500 mL of saline solution for peritoneal effusions.

**Results:**

Participants were followed up for a median time of 13.6 month. A total of 11 cases, 7 with pleural effusions and 4 with peritoneal effusions achieved a complete response (CR), and 20 cases (12 pleural effusions and 8 peritoneal effusions) had a partial response (PR). The overall response rate is 64.6%. Patients' quality of life, assessed by using Karnofsky performance scale (KPS) scores, was improved by an average of 26.4. The one-year of overall survival rate was 54.2% with a median survival time of 12.5 months. There were no serious side effects observed except for self-limited fever found in 79.8% of the cases.

**Conclusions:**

Intra-cavity infusion of rAd-*p53* is an effective and safe treatment for the patients with malignant pleural or peritoneal effusions, especially for those patients who can't tolerate the standard treatments.

## Introduction

Malignant pleural or peritoneal effusion is one of common clinical presentations of malignant tumor in advanced stage. Persistent large amounts of effusions seriously diminish patients' quality of life and even threaten their life. Once the effusion developed it is difficult to control. Many effusions fail to respond to the standard treatments for the primary tumor. Although there are many effective local treatments available for malignant effusions, not every treatment works for every patient. Most these treatments are palliative and have adverse effects or complications. Chemically induced pleurodesis, especially using talc as an intrapleural agent, is very effective for controlling malignant pleural effusions. However, this procedure has narrow indications and also associated with up to 10% of acute respiratory failure^[1]^. Thoracenteses or abdominocentesis is a necessary procedure for diagnosis and immediate relief of symptoms. But frequent and repeated the procedure can induce many complications, such as infection, and protein depletion^[2]^. Intra-pleural or abdominal chemotherapy was believed to treat the underlying malignancy as well as the effusion. However, clinical data indicated the intracavity chemotherapy has a limited role in treatment of both effusion and primary malignant tumor. The overall response rate of this local chemotherapy is around 40%-60%^[3]^. Many patients with advanced malignances can't tolerate the adverse effects of intracavity chemotherapy.

*P53* gene has been well studied and is one of most important tumor suppressing gene. It has multiple anti-tumor functions including blocking tumor cell cycle, inducing tumor cell apoptosis, inhibiting tumor angiogenesis and sensitizing tumor to chemo- and radio-therapy^[4-9]^. Recombinant adenoviral human *p53* gene (rAd-*p53*) has been shown to be effective for many types of solid malignant tumors^[10-13]^. The rAd-*p53* also demonstrated an excellent safe profile. It is well tolerated by the patients with advanced malignance or in poor condition. Objective of this study is to investigate of efficacy and safety of rAd-*p53* in treatment of malignant pleural or peritoneal effusion.

## Materials and methods

The study was proved by our hospital ethics committee but not registered in an international clinical trial registry platform. All the enrolled patients signed a consent form.

### *Patients* 

From February of 2008 to July of 2012, a total of 48 patients, 29 males and 19 females with an average age of 61.2 years old, who were satisfied with the following inclusion criteria, were recruited in this study. The inclusion criteria are: histopathologically diagnosed primary cancer in stage of Ⅲ or Ⅳ with pleural or peritoneal effusion; the effusion failed to respond to the standard treatment for primary tumor; over 18 years old; with normal hemogram and blood coagulation tests; understanding and signing the informed consent form. The patients' characteristics and disease stage are summarized in Table 1.

**1:** Patients' characteristics

Characteristics	Statistics
Age (year)	61.2±22.4 (41-80)
Gender	
Male	29 (60.4%)
Female	19 (39.6%)
Stage	
Ⅲ	12 (25.0%)
Ⅳ	36 (75.0%)

Twenty-seven patients had a malignant pleural effusion caused by lung cancer (18 cases), breast cancer (4 cases), lymphoma (2 cases), esophageal cancer (1 case), thymus cancers (1 case), and metastatic malignant tumors (1 case); and 21 patients had a malignant peritoneal effusions caused by ovarian cancer (9 cases), gastric cancer (4 cases), pancreatic cancer (2 cases), bile duct cancer (2 cases), rectal cancer (1 cases), appendiceal mucinous adenocarcinoma (1 case), pseudomyxoma (1 case) and retroperitoneal adenocarcinoma (1 case).

### *Treatments* 

RAd-*p53* was from Shenzhen Sibiono Genetech Co., Ltd. (19 1^st^ Science & Technology Middle Road, Shenzhen, Guanddong Province 518087, China). After draining most of fluids using standard thoracenteses or abdominocentesis, these patients received intra-cavity infusion of rAd-*p53* once a week for 4 weeks, at dose of 2×10^12^ viral particles (VP) diluted into 200 mL of saline solution for pleural effusions, and 4×10^12^ VP diluted into 500 mL of saline solution for peritoneal effusions.

### *Assessments* 

All these patients were followed up every 1 month to assess effectiveness, quality of life, and safety. Routine physical examination, lab tests including hemogram, liver and kidney function tests, and morphologic imaging tests were performed during each follow-up. The response of rAd-*p53* in treatment of malignant pleural or peritoneal effusions was assessed using the WHO criteria (1979). The CR was defined as the complete disappearance of pleural or peritoneal fluid and negative cytologic findings for >4 weeks, and the PR is defined as a decrease over 50% of the fluid without the need of drainage for >4 weeks. Overall survival was estimated using *Kaplan*-*Meier* Method. Quality of life was assessed using Karnofsky performance scale (KPS) scores with a score from 0 to 100, where 0 is dead and 100 is completely normal^[10]^. The safety profile was assessed by recording adverse events, physical examination, lab and imaging test.

## Results

Participants were followed up for a median time of 13.6 months. A total of 11 cases, 7 with pleural effusions and 4 with peritoneal effusions achieved a complete response (CR), and 20 cases (12 pleural effusions and 8 peritoneal effusions) had a partial response (PR). All other patients responded the treatment but were either in short time or not able to satisfy the CR or PR criteria. The overall response rate is 64.6%. Patients' quality of life, assessed by using Karnofsky performance scale (KPS) scores, was improved by mean of 15, with a statistically significant difference comparing the scores of post-treatment to the scores of pre-treatment. Table 2 shows the detail summary of KPS scores. The one-year of overall survival rate was 54.2% (26/48) with a median survival time of 12.5 months. The *Kaplan*-*Meier* survival curve is shown in Figure 1. There were no serious side effects observed except for self-limited fever found in 81.3% (39/48) of the cases. The fever occurred at 2-12 hours after the infusion of rAd-*p53*. For the most cases (31/48, 64.6%) fever was under 38℃ and needed no treatment. Eight cases with a fever of 38.2 ℃-40.7 ℃ were treated by physical cooling or by intra-venous infusion of 5 mg of dexamethasone.

**2:** Analysis of the KPS scores before and after treatment

KPS scores of pre-treatment (Mean±SD)	KPS scores of post-treatment (Mean±SD)	Difference (Mean±SD) *P*
58.1±12.00	73.1±9.03	15.0±12.03 *P* < 0.001

**1:**
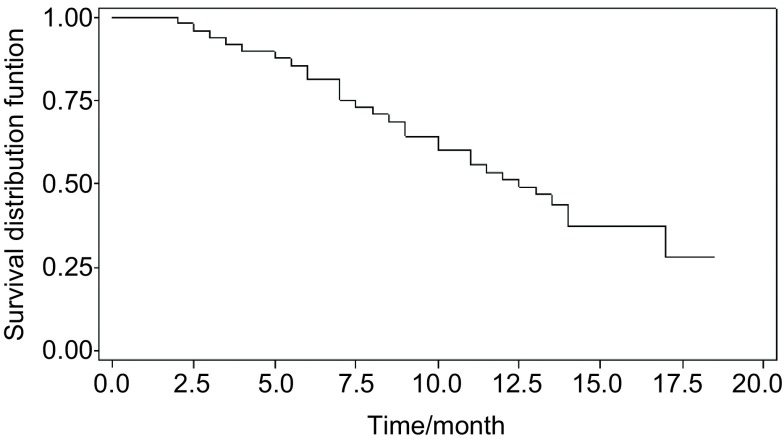
Survival curve of patients with malignant effusions

## Discussion

Malignant pleural or peritoneal effusion is one of common clinical presentations of malignant tumors in advanced stage, and indicates dissemination of the primary tumor. Once the effusions developed they are difficult to control. Some malignant effusions respond to the treatments for primary tumor. But for the most cases local therapy is required. There are many local treatment options and no single therapy is appropriate for all patients. In this study, all the patients failed to respond to the treatments for the primary tumor. Thus, a local method is required to control the effusions. Most of these patients were in a very poor condition and were not able to tolerate or couldn't agree to accept more invasive treatment. A less invasive and safe method is appropriate for these patients.

*P53* is one of most important tumor-suppressor genes and has multiple anti-cancer functions including inhibiting cell cycle, inducing apoptosis, inhibiting tumor angiogenesis, and sensitizing chemo- and radio-therapy. Its anti-tumor activities were shown in the cell-based study, tumor animal models, and clinical studies^[14, 15]^. Several reports demonstrated that rAd-*p53* is effective for some solid malignant tumors. The rAd-*p53* also has an excellent safe profile. Its common adverse effects are self-limited fever, shiver and cold-like symptoms^[10-13]^. Since its clinical application, no serious adverse effects have been reported. In this study, we achieved an overall response rate of 64.6% using intra-cavity infusion of rAd-*p53*. Patients' quality of life, assessed by using KPS scores, was significantly improved. Intra-cavity infusion of rAd-*p53* also demonstrated a high safety profile and well tolerated by all the patients. A mild or moderate self-limited fever was commonly observed side effects. There were no serious side effects observed.

All the patients in this study had a malignant tumor in the stage Ⅲ or Ⅳ with malignant pleural or peritoneal effusions. Their pleural or peritoneal effusions failed to respond the standard treatments for the primary tumors. The option of treatments for these cavity effusions was limited. According to its efficacious and safe profiles, rAd-*p53* is a reasonable treatment for these cavity effusions. The short-term efficacies in this study were favorable, but a controlled study with a long-term of follow-up is required to confirm the treatment effects of rAd-*p53* for malignant pleural or peritoneal effusions.

In conclusion, rAd-*p53* is a potential favorite treatment for the malignant pleural or peritoneal effusions, especially for the patients with a malignant tumor in advanced stage and in a poor condition.
